# Selections

**Published:** 1894-04

**Authors:** 


### Selections.

### THE DEFECT IN THE DENTIST.

The dentists of America are the best in the world, but they are, nevertheless, away behind the mark. We appreciate their ingenuity, their skill, their drills and dams, their composite fillings, and artificial teeth. They make the best kind of golden crowns, but do not deserve to wear them. This is because they do not sterilize themselves, or teach their patients how to prevent caries. They are wonderful patchers-up of things half gone, but they do not show how to prevent the going.

There is hardly a dentist (there are a few) who works in an aseptic way or uses aseptic instruments. Some do not know any better, some do not think it necessary. But both are wrong; the one class should learn, the other class should think. How many dentists can tell surely when they are dealing with a syphilitic mouth? Yet if such a mouth is being treated, we do not believe that any one would like to be the next patient, though the dentist should first wash his hands.

The mouth is a rich culture-ground for microbes, and caries, it is taught, is a microbic disease. Yet the dentist is content to fill the teeth, then disburse a bottle of his own special mixture of chalk, orris, and wintergreen, and tell his patient to brush the teeth *vis die.* The ordinary tooth brushing is entirely ineffective in preventing caries or disinfecting the mouth. For this purpose, a genuinely antiseptic wash is needed, and this should be used for a considerable time. Just what this wash in powder should be, and just how long it should be used, is one of the things the dentist should find out. Let the American dentist devise a way of making the teeth and gums aseptic, and preventing caries. He will then be greater than if he had invented a new filling or a novel drill, or transplanted a whole jaw-full of teeth.—*New York Medical Record.*

